# Environmental risk factors associated with respiratory diseases in children with socioeconomic disadvantage

**DOI:** 10.1016/j.heliyon.2021.e06820

**Published:** 2021-04-22

**Authors:** Javier Cortes-Ramirez, Juan D. Wilches-Vega, Olga M. Paris-Pineda, J.E. Rod, Lkhagvadulam Ayurzana, Peter D. Sly

**Affiliations:** a School of Public Health and Social Work, Queensland University of Technology, Brisbane, Australia; b Children's Health and Environment Program, University of Queensland, Brisbane, Australia; c Faculty of Health, University of Santander, Cúcuta, Colombia; d Centre for Accident Research and Road Safety Queensland (CARRS-Q), Queensland University of Technology (QUT), Brisbane, Queensland, Australia; e Institute of Health and Biomedical Innovation Queensland University of Technology (QUT), Brisbane, Queensland, Australia; f School of Public Health, University of Queensland, Brisbane, Australia

**Keywords:** Environmental exposures, Household air pollution, Industrial pollution, Particulate matter, Systematic review, Tobacco smoking

## Abstract

Children are more vulnerable to environmental exposures determinant of respiratory diseases due to their dynamic developmental physiology. Whereas social determinants of health are also associated with a higher risk of these diseases in children exposed to environmental risk factors, most studies incorporate them as covariates in the statistical analysis rather than focusing on specific vulnerable populations. In this study a systematic review searched and selected studies of respiratory diseases in children with socioeconomic disadvantage to identify the environmental risk factors associated with these diseases. The review followed the PRISMA protocol to identify eleven eligible studies of children with socioeconomic conditions that included low income and low socioeconomic status, overcrowding, adults with low education level and Indigenous status. Infectious respiratory diseases, asthma, rhinitis and mortality due to respiratory diseases were associated with risk factors such as biomass fuel use, tobacco smoking, particulate matter, coal dust and other pollutants including ozone, nitrogen dioxide and sulphur dioxide. The most common associations were between respiratory infections and household air pollution and asthma with indoor and outdoor air pollution. The findings support previous reports on these associations and suggest that specific vulnerabilities such as indigenous children and living with adults with low socioeconomic status and education level increase the risk of respiratory diseases. These populations can be given special attention to prioritize public health interventions to lower the burden of disease of respiratory diseases in children.

## 1 Introduction

Adverse environmental exposures are determinants of a higher risk of respiratory diseases, with indoor and ambient air pollution accounting for 7 million deaths globally (World Health Organization, 2018). The mortality and burden of these diseases impact more on vulnerable populations, particularly children who have higher exposure to air pollutants due to their dynamic developmental physiology and longer life expectancy (Sly and Flack, 2008). Indoor tobacco smoke exposure is associated with respiratory symptoms and increasing incidence of respiratory diseases in children, including asthma, rhinitis and infections of the respiratory tract (Zhuge et al., 2020). Exposure to particulate matter (PM) in ambient air increases the risk of acute lower respiratory infections, pneumonia and asthma in children (Davila Cordova et al., 2020). Other ambient air pollutant such as sulphur dioxide, ozone, nitrogen dioxide, and carbon monoxide have been associated with hospitalizations due to pneumonia in children under five years (Nhung et al., 2017). The environmental exposure to PM, second-hand smoke and ozone as well as formaldehyde, dampness and lead have the largest impact on the environmental burden of disease in populations aged below 18 years in European countries. These exposures were responsible for 210,777 disability-adjusted life years (DALYs) in 2015, with the highest DALYs per year in 1-year old children (Rojas-Rueda et al., 2019). Although environmental factors determine major risks to children's respiratory health worldwide, the most affected are middle and low income countries (Saleh et al., 2020), which highlights the detrimental impact of socioeconomic factors on morbidity and mortality due to respiratory diseases.

Social determinants of health shape the distribution of morbidity in the childhood period, due to unequal access to health care and services; and the greater burden of health inequities in socioeconomically disadvantaged populations plays an important role in the development of respiratory diseases. Children from poor socioeconomic backgrounds have a higher risk of respiratory diseases including asthma (Foronda et al., 2020) and acute respiratory infections (Moore et al., 2019). These differences can be related to reduced respiratory function identified in socioeconomically disadvantaged children and adolescents (Rocha et al., 2019). The plausibility of respiratory health outcomes associated with detrimental socioeconomic circumstances is often compounded by the occurrence of other determinants, especially environmental risk factors such as household air pollution (Simkovich et al., 2019). However, most epidemiological studies analyse the association of respiratory diseases and environmental risk factors for socioeconomic segments rather than identifying specific attributes of socioeconomic disadvantage such as low education or ethnicity. For example, analyses at the regional level classify countries by income threshold (i.e. low, middle, and high income) and country-specific studies use socioeconomic status without considering specific vulnerable populations such as Indigenous or crowded communities. This restricts the understanding of the risk of respiratory health outcomes in children in social vulnerability due to the imbalance of health inequalities between and within countries (Marmot et al., 2008). The aim of this study is to systematically review the scientific literature to identify associations between respiratory diseases and environmental risk factors focusing on children with specific socioeconomic disadvantages.

## 2 Methods

A systematic review was undertaken following the PRISMA protocol (Moher et al., 2009). The Systematic search included studies in Pubmed, Embase, Lilacs and Scielo, published until 01/10/2019. The search strategy included combinations of the terms: child, respiratory, risk factor and environment (search strategy code for each database in the appendix).

### 2.1 Selection criteria

The eligible studies had to measure the association of environmental risk factors with respiratory health outcomes in children characterized by socioeconomic disadvantage. Environmental risk factors included agents or hazards in air, soil and or water. Socioeconomic disadvantage included low-income and poverty, overcrowding, low education level or illiteracy, and Indigenous status.

### 2.2 Exclusion criteria

Reviews articles were excluded. Studies reported in languages different than English or Spanish (language proficiency of the authors) were excluded.

### 2.3 Data collection

A data form pre-piloted with 10% of the eligible studies (i.e. selected for full-text reading), was designed to extract data on the analytical methods, population characteristics, measures of risk and covariates used in the selected studies.

### 2.4 Synthesis analysis and quality assessment of the selected studies

The studies were categorized according to the respiratory diseases and the environmental risk factors and measures of risk estimated (e.g. relative risk, odds ratio, proportional excess of risk). A critical appraisal of the studies to estimate the risk of bias was done using the Newcastle-Ottawa Scale (NOS) (Wells et al., 2016). The scale assessed the selection of cases and controls and the comparability of cases and controls on the basis of the design or analysis, and the assessment of exposures with a maximum score of 9 points. A low risk was considered for studies that reached 7 or more points, moderate risk: 5 to 6 points and high risk for scores less than 5.

Two independent researchers carried out the literature search and articles selection process. Discrepancies were discussed with a third researcher until consensus was reached. Data extraction and synthesis were implemented between November 2019 and February 2020.

## 3 Results

**Figure 1:**
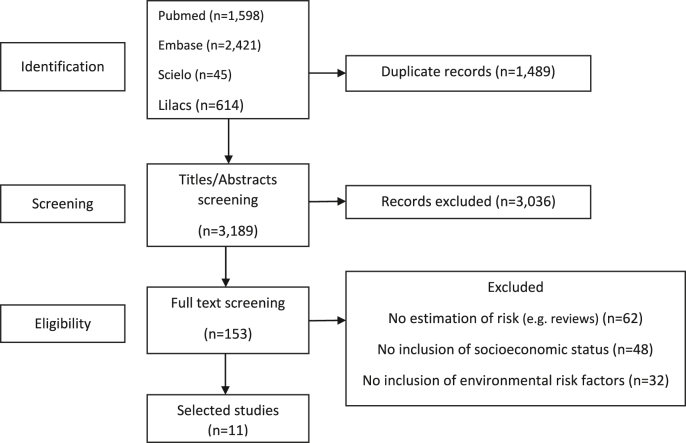
Retrieval flowchart and selection of articles.

A total of 4678 articles were identified in the search strategy. After exclusion of duplicates and screening of titles and abstracts, 153 articles were eligible for independent full-text reading from which 11 articles were selected for the review (Figure 1).

All of the selected articles were published between 2002 and 2017, except one study conducted in 1994 (Brabin et al., 1994). Five studies (45%) were done in Latin-American countries (Amarillo and Carreras, 2012; Bautista et al., 2009; Cardoso et al., 2013; Nascimento-Carvalho et al., 2002; Romieu et al., 2004); 2 studies (18%) in Asian countries (Doshi et al., 2015; Le et al., 2012), one study (9%) in Africa (Mustapha et al., 2011), one study (9%) in the United Kingdom (Brabin et al., 1994) and one study (9%) in the United States of America (Goodman et al., 2017). All of the studies implemented regression analyses to estimating risk, except one study that used a Pearson's chi-square test (Nascimento-Carvalho et al., 2002). All of the studies had a low risk of bias according to the NOS. Table 1 shows the risk of bias by domain for the selected studies.

**Table 1:** Risk of bias in the selected studies using the Newcastle-Ottawa scale.

	Criterion	Papers that reached a point
Selection	case definition adequate	11/11 (100%)
	Representativeness of cases	11/11 (100%)
	Selection of Controls	10/11 (90%)
	Definition of Controls	11/11 (100%)
Comparability	Comparability of cases and controls (at least one point)	11/11 (100%)
Exposure	Ascertainment of exposure	8/10 (73%)
	Same method of ascertainment for cases and controls	11/11 (100%)
	same non-response rate (case/controls)	11/11 (100%)

Socioeconomic indexes or measures of income were used as indicators of socioeconomic disadvantage in 7 (64%) of the studies (Bautista et al., 2009; Goodman et al., 2017; Le et al., 2012; Mustapha et al., 2011; Nascimento-Carvalho et al., 2002; Romieu et al., 2004; Silfeler et al., 2012). Other indicators of socioeconomic disadvantage included education level (Amarillo and Carreras, 2012); major housing and unemployment problems (Brabin et al., 1994); Indigenous status (Cardoso et al., 2013) and overcrowding (Doshi et al., 2015). All of the studies included health outcomes of morbidity except one study of mortality due to respiratory diseases (Romieu et al., 2004). Respiratory infections (including upper and lower tract respiratory infections, influenza and pneumonia) were the health outcome in seven (64%) of the studies, and asthma was investigated in three (27%) of the studies. The risk factors associated with respiratory diseases in the selected studies were indoor biomass fuel use; particulate matter (PM_10,_ PM_2.5,_ PM_4_), tobacco, coal dust, ozone (O_3_), nitrogen dioxide (NO_2_) and sulphur dioxide (SO_2_) (Table 2).

**Table 2:** Characteristics of the studies selected in the review.

Author(s) (year)	Country	Analytical method	Type of SE disadvantage	Environmental risk factor(s)	Health outcome(s)
Amarillo and Carreras (2012)	Argentina	Generalised additive regression	Adults's education level	PM_10_	URTI, LRTI
Bautista et al. (2009)	Dominican Republic	Multiple logistic regression	Low income	Indoor biomass fuel (charcoal) and PM_4_	LRTI
Brabin et al. (1994)	United Kingdom	Multiple logistic regression	Major housing and unemployment problems	Coal dust	Asthma; wheezing
Cardoso et al. (2013)	Brazil	Hierarchical multivariate logistic regression	Guarani Indigenous children	Indoor biomass fuel	LRTI
Doshi et al. (2015)	Bangladesh	Multivariate regression	Overcrowding	Indoor biomass fuel	Influenza
Goodman et al. (2017)	USA	Generalised additive regression	Low SES	Indoor PM_2.5_; O_3_	Asthma
Le et al. (2012)	Vietnam	Poisson regression	Poverty (SE index)	PM_10_; O_3_; NO_2_; SO_2_	Influenza
Mustapha et al. (2011)	Nigeria	Multilevel logistic regression	Low SES	Indoor biomass fuel	Asthma; rhinitis
Nascimento-Carvalho et al. (2002)	Brazil	Pearson's chi-squared test	Low SES	Tobacco	Pneumonia
Romieu et al. (2004)	Mexico	Multiple Logistic regression	Low SES	PM_10_	Respiratory-related mortality
Silfeler et al. (2012)	Turkey	Logistic regression	Low SES	Tobacco	LRTI

Notes. SE: Socioeconomic, SES: Socioeconomic status, URTI: Upper respiratory tract infection; LRTI: lower respiratory tract infection.

Table 3 shows the estimates of risk for each of the respiratory diseases studied, in relation to the environmental risk factors included. All of the studies found a positive association (increased risk) between environmental risk factors and respiratory diseases with exception of three studies that found; lower risk of asthma and rhinitis in children exposed to indoor biomass use (Mustapha et al., 2011), lower excess of risk of influenza in children exposed to PM_10_ in the wet season, and lower risk of asthma in children exposed to O_3_ (Le et al., 2012) -although these estimates were not statistically significant. All of the studies of children with lower respiratory tract infections found a significant association of these diseases with environmental risk factors (Amarillo and Carreras, 2012; Bautista et al., 2009; Cardoso et al., 2013; Silfeler et al., 2012). Likewise, the two studies that included tobacco (indoor smoking) as a risk factor, found a significant association with lower respiratory tract infections (Silfeler et al., 2012) and pneumonia (Nascimento-Carvalho et al., 2002), respectively. PM_10_ was associated with an increased risk of lower respiratory tract infections, influenza, upper respiratory tract infections and mortality associated with respiratory diseases. These associations, however, were significant only in one study (Amarillo and Carreras, 2012). There was an increased risk of asthma and respiratory symptoms in children exposed to coal dust, PM_2.5_ and O_3_, although these associations did not reach statistical significance (Brabin et al., 1994; Goodman et al., 2017). There was no significant but consistent positive association between the exposure to higher levels of O_3_, NO_2_ and SO_2_ and a higher risk of asthma and influenza in the wet season (Goodman et al., 2017; Le et al., 2012) and lower risk of influenza in the dry season (Le et al., 2012).

**Table 3:** Risk of respiratory diseases in children with socioeconomic disadvantage exposed to environmental risk factors.

	indoor biomass fuel use	smoking	PM10	PM25	coal dust	indoor PM4	O_3_	SO_2_	NO_2_
lower respiratory tract infection	OR = 1.38 (1.06–1.81) Bautista et al. (2009) OR = 3.08 (1.28–7.45) p < 0.013 Cardoso et al. (2013)	RR = 2.63 (1.30–2.09) Silfeler et al. (2012)	%exR = 5.61 (4.98–6.24) Amarillo and Carreras (2012)			OR = 1.17 (1.02–1.34) Bautista et al. (2009)			
Influenza	OR = 1.77 (1.18–2.67) p < 0.006 Doshi et al. (2015)		%exR (dry season) = 3.43 (-11.88 to 21.41)/%exR (wet season) = -0.94 (-14.54 to 14.82) Le et al. (2012)				%exR (dry season) = 3.43 (-11.88–21.41)/%exR (wet season) = -0.94 (-14.54–14.82) Le et al. (2012)	%exR (dry season) = 3.43 (-11.88–21.41)/%exR (wet season) = -0.94 (-14.54–14.82) Le et al. (2012)	NO2 exR (dry season) = 17.17 (-39.64–127.45)/NO2 exR (wet season) = -21.78 (-52.69–29.34) Le et al. (2012)
Asthma	OR = 0.26 (0.02–4.15) Mustapha et al. (2011)			RR = 1.0103 (0.97–1.04) <6 years/RR = 1.034 (0.99–1.07) 6–18 years Goodman et al. (2017)	OR = 1.25 (0.88–1.76) Brabin et al. (1994)		RR = 0.99 (0.97–1.01) <6 years/RR = 1.02 (0.99–1.04) 6–18 years Goodman et al. (2017)		
Pneumonia		% risk severity = 48% (vs 31% controls). p < 0.0001 Nascimento-Carvalho et al. (2002)							
Upper respiratory tract infection			%exR = 4.59 (4.13–5.05) Amarillo and Carreras (2012)						
Wheezing					OR = 1.25 (0.88–1.76) Brabin et al. (1994)				
Rhinitis	OR = 0.26 (0.02–4.15) Mustapha et al. (2011)								
Resp. mortality			OR = 1.61 (0.97–2.66) Romieu et al. (2004)						

Notes. OR: Odds ratio; RR: Relative risk; %exR: Proportional excess of risk; PM: particulate matter; O_3_: ozone; NO_2_: Nitrogen dioxide; SO_20_: Sulphur dioxide.

## 4 Discussion

This study found consistent and significant associations between environmental risk factors and respiratory diseases, especially infections of the lower and upper respiratory tract, pneumonia and influenza, in children with socioeconomic disadvantage. The most important factors contributing to the higher risk of these diseases were smoking and indoor use of biomass fuel as well as particulate matter. Other environmental risk factors such as coal dust, ozone, oxides of nitrogen and sulphur dioxides where associated with respiratory diseases, especially influenza, asthma and respiratory symptoms. Children affected by overcrowding, Indigenous children and children with adults with low education level or socioeconomic status had higher mortality due to respiratory diseases and higher risk of respiratory infections and asthma. An increased risk of respiratory infections related to environmental risk factors has been established in multiple studies of young children (Admasie et al., 2018; Haerskjold et al., 2016; Tazinya et al., 2018). We identified that children with socioeconomic vulnerability have a higher risk of infections of the respiratory tract associated with indoor and outdoor air pollution, which is consistent with the findings of previous research in children with these environmental exposures. Exposures to ambient and household air pollution can be responsible for altered immunological responses that increase the susceptibility of the respiratory system to microorganisms. It has been established that early life and in-utero exposure to tobacco smoke impact the immune system development in association with inadequate cytokine production and changes in T-cells numbers that determine a poor reaction to invasive pathogens (MacGillivray and Kollmann, 2014). While there is a well-known relationship between indoor air pollution and respiratory infections, children under socioeconomic stress have greater risk of morbidity due to respiratory inflammatory diseases (Foley et al., 2019). These associations explain the higher risk of respiratory infections in socioeconomically vulnerable children and can be related to other conditions such as allergic responses of the respiratory system and asthma.

Our findings include a positive association of indoor and outdoor air pollution with asthma and rhinitis, which represent a significant contribution to the burden of disease in children. These associations are commonly found in studies of children in developing regions where socioeconomic disadvantage has a significant impact on the children's respiratory morbidity (Asher and Pearce, 2014). The strong association of respiratory morbidity with socioeconomic disadvantage is found in multiple studies implemented in developing countries which may seem like a geographically grouped issue (Shi et al., 2017). However, we identified the increased risk in studies in diverse socioeconomic contexts and identified specific socially vulnerable conditions such as poverty and socioeconomic status, in low- and high-income countries, which highlights the role of social determinants of health within countries. Most studies incorporated adjustment for socioeconomic indexes at a group level as a proxy to determine the impact of social determinants of health on health outcomes in children. Whereas we identified common socioeconomic risk factors for respiratory diseases addressed in other studies such as low income and socioeconomic status, we also found that indigenous children and children living in overcrowding or with adults with low education level have higher risk of infectious respiratory diseases. This highlights the importance of studying distinctive populations with socioeconomic risk, independently from broader categorizations of socioeconomic status, to have a better understanding of the environmental risk factors of respiratory diseases in children.

This review identified that high concentration of pollutants in air including particulate matter, coal dust and ozone, was associated with respiratory diseases in children at socioeconomic risk. Particulate matter can deposit in different sections of the respiratory tract depending on particle size and solubility, inducing an immune response characterized by the development of inflammation and oxidative stress. This response, in turn, increases the vulnerability to asthma and infectious and chronic respiratory diseases. An increased risk of morbidity due to respiratory outcomes in children in areas with lower socioeconomic index linked to higher concentrations of particulate matter has been found in high and middle income countries (Yap et al., 2013; Zhou et al., 2018). Our findings are consistent with these results with a higher risk of respiratory infections and asthma in children living with adults with low education and socioeconomic index. We also found that poor socioeconomic conditions were associated with exposure to other pollutants such as coal dust that are prevalent in populations with major housing and unemployment problems. These concur with previous research that suggest that poor populations in proximity to extractive industries are at higher risk of respiratory diseases due to higher concentration of air pollutants in these areas (Cortes-Ramirez et al., 2018). On the other hand, higher ambient levels of ozone from photochemical reactions of primary precursors such as nitrogen oxides and volatile organic compounds from industrial activities are associated with more frequent emergency department visits due to acute respiratory infections, pneumonia and asthma in children (Strosnider et al., 2019). In our findings there was an increased risk of influenza in the dry season in children with low socioeconomic index living in industrial areas (Le et al., 2012) which corroborates the risk that highly polluting industrial activities pose to the children's respiratory health.

Other ambient air pollutants identified in this study include sulfur oxides and oxides of nitrogen which are common by-products of industrial fossil fuel combustion (e.g. coal generation of electricity) and motor vehicle emissions. Changes in airways responsiveness, bronchoconstriction and detrimental lung function prompted by these agents are mostly associated with a higher risk of asthma and respiratory infections (US Environmental Protection Agency, 2016). Although only one study in our review identified an increased risk of influenza in children exposed to higher ambient air concentration of SO_2_ and NO_2_ (Le et al., 2012) recent research highlights the risk of indoor concentration of these and other pollutants such as particulate matter (Huang et al., 2019). Even small indoor concentrations of these substances can have a significant impact on the respiratory system because people spend about 90 percent of their time indoor. This is especially relevant for children living in socioeconomic conditions that determine the use of fossil fuel stoves for cooking in poor ventilated spaces (Huang et al., 2019). The impact of indoor fuel use on children's respiratory health can be reduced with cost effective interventions such as the incorporation of stoves with chimneys or ethanol stoves and education programs for modifying users behavior (Pope et al., 2017). However a significant proportion of the world population are still at risk because of household air pollution (World Health Organization, 2018). The above results combined establish the importance of identifying populations at risk to prioritize public health interventions. The use of consistent evidence on children from socioeconomic and or demographic groups with higher vulnerability to respiratory diseases is a useful tool to decrease the overwhelming burden of disease associated with exposures to air pollutants.

### 4.1 Limitations

There was a small number of selected articles in this review due to a paucity of studies on environmental risk factors associated with respiratory morbidity with focus on specific socioeconomic disadvantages. This is related to the inclusion of socioeconomic indicators applied to large populations such as socioeconomic status indexes in most studies, which marginalizes the role of specific socioeconomic conditions as risk determinants. Our search strategy included articles published in English and Spanish that allowed the inclusion of studies of specific populations such as Indigenous children in Latin America and children with adults with low education. However, studies in other languages could have assessed children with other socioeconomic attributes and we identified only a discrete list of specific socioeconomic risk factors. In addition, other socioeconomic disadvantages in potentially more vulnerable populations such as rurality, lack of health services and nonmedical treatment of respiratory diseases could not be identified in the studies selected. We focused the search strategy on studies with measures of the association of risk factors with respiratory diseases which allowed the analysis of quantitative findings but provided a reduced number of eligible studies and a small list of socioeconomic conditions identified.

A further issue is that the studies included produced diverse estimates of risk, including relative risk odds ratio and proportion of excess of risk. Although most studies used linear regression models for estimating risk, the diverse methodological approaches and measures estimated, complicated the identification of quantitative trends of risk for respiratory diseases in children with somewhat similar socioeconomic and environmental risk factors. We were able to identify each environmental risk factor associated with specific health outcomes although a larger sample of selected studies would be required to determine risk trends for specific environmental risk factors.

## 5 Conclusions

Environmental risk factors including the use of biomass fuel for cooking, tobacco smoking, particulate matter, and industrial air pollutants are determinants of respiratory diseases in children whose risk increases depending on their socioeconomic conditions. The higher risk is not restricted to low-income countries and is present in low-income and disadvantaged populations within middle- and high-income countries. Special attention should be given to Indigenous children and children with adults with low socioeconomic status and education level. Further research is warranted to identify other specific socially vulnerable populations and the trends of risk at a regional level.

## Declarations

### Author contribution statement

Javier Cortes-Ramirez: Conceived and designed the experiments; Analyzed and interpreted the data; Contributed reagents, materials, analysis tools or data; Wrote the paper.

Juan D. Wilches-Vega, Olga M. Paris-Pineda, J E. Rod, Lkhagvadulam Ayurzan and Peter D. Sly: Analyzed and interpreted the data; Wrote the paper.

### Funding statement

This research did not receive any specific grant from funding agencies in the public, commercial, or not-for-profit sectors.

### Data availability statement

Data included in article/supplementary material/referenced in article.

### Declaration of interests statement

The authors declare no conflict of interest.

### Additional information

No additional information is available for this paper.

## Appendix

**Search strategy code for each database:**

**Pubmed**: (("Child"[Mesh]) AND (((risk∗[Title/Abstract]) AND factor∗[Title/Abstract]))) AND respirator∗[Title].

**Embase**: 'child'/exp AND (risk∗:ab,ti AND factor∗:ab,ti) AND respirator∗:ti

**Lilacs**: (mh:(factor∗)) AND (mh:(ambient∗)) AND (tw:(niñ∗)) AND (tw:(respirator∗))

**Scielo**: ((ti:(ambient∗)) OR (ab:(ambient∗))) AND ((ti:(factor∗)) OR (ab:(factor∗))) AND ((ti:(niñ∗)) OR (ab:(niñ∗))) AND ((ti:(respirator∗)) OR (ab:(respirator∗)))
